# Referral from secondary care and to aftercare in a tertiary care university hospital in Japan

**DOI:** 10.1186/1472-6963-6-11

**Published:** 2006-02-17

**Authors:** Shin-ichi Toyabe, Akazawa Kouhei

**Affiliations:** 1 Department of Medical Informatics, Niigata University Medical and Dental Hospital, Asahimachi-dori 1-754, Niigata 951-8520, Japan

## Abstract

**Background:**

In Japan, all citizens are covered by the national insurance system in which universal free access to healthcare services is promised to everybody. There are no general physicians or gatekeepers in the Japanese healthcare system.

**Methods:**

We studied the pattern of referral of inpatients from secondary care hospitals to a tertiary care university hospital and the reverse referral under the situations using a geographic information system (GIS), taking paediatric inpatients as an example.

**Results:**

The results showed that 61.2% of the patients were directly admitted to the hospital without referral from other hospitals or clinics and that 82.8% of the inpatients were referred to the outpatient department of the hospital to which they had been admitted. GIS analysis for the inpatients service area showed the hospital functions as both a secondary care hospital and tertiary care hospital. Patients who lived near the hospital tended to be admitted directly to the hospital, and patients who lived far from the hospital tended to utilize the hospital as a tertiary care provider. There were territorial disputes with other secondary care hospitals. To estimate spatial differences in referral to aftercare, we analyzed the spatial distribution of inpatients with focus on their length of hospital stay (LOS). GIS analysis revealed apparent foci of patients with long LOS and those with low LOS.

**Conclusion:**

These results suggest that the function of university hospital in Japan is unspecialized and that the referral route from the university hospital to aftercare is also unequipped.

## Background

In Japan, all citizens are covered by the national insurance system in which universal free access to healthcare services is promised to every people. There are no general physicians or ''gatekeepers'' in the Japanese healthcare system. Patients in Japan can freely choose between going to a physician's office or a hospital [1]. Hospitals are popular because of their prestige and high quality of care. On the other hand, there is no clear differentiation between large and small or public and private hospitals or between hospitals and private practices in the healthcare system in Japan [1]. Functional differences between hospitals and nursing homes for the elderly are also not clear despite of the introduction of long-term care insurance [2]. Hospitals often function as nursing homes for the elderly, which is one of the main reasons for excessive long hospital length of stay (LOS) in Japan. Actually, LOS is very long in Japan compared with other Organization for Economic Co-operation and Development (OECD) countries [3], particularly for the elderly.

The government has tried to address the issue of undifferentiated functions of health care facilities. Fees for referral were introduced in 1988 and outpatient consultation fees for university hospitals were increased if more than 30 percent of their new ambulatory care patients came with referrals [4]. Since 1996, patients who visit a large hospital without referral from a primary care physician have had to pay an extra charge [5]. In 2003, an inclusive payment system for acute inpatient care was introduced in university hospitals [6]. They are based on the policy to facilitate functional differentiation of the health care facilities. However, little is known about the actual situations of referral from the secondary care to tertiary care university hospitals and the reverse referral in Japan. We studied the pattern of referral form secondary care to tertiary care university hospital and the reverse referral in Japan, taking paediatric inpatients as an example.

## Methods

### Data

In 2004, Niigata University Hospital had 810 inpatient beds, 23 clinical departments and medical staff of 818. The service area of the hospital as a tertiary care hospital covers all districts in Niigata Prefecture, which has a population of 2,400,000. There are 34 paediatric inpatient beds in ward for children. These beds do not include beds for paediatric surgery patients. The size of the hospital is typical for a Japanese university hospital.

Patient data were analyzed for all children and adolescents under fifteen years of age who had received inpatient care at Niigata University Hospital during the period from April 2004 to March 2005. Information on paediatric inpatients under 15 years of age was collected. Data on patient age, sex, residential address, route of referral to Niigata University Hospital, discharge diagnosis classified by Japanese case mix classification for inpatients (diagnosis procedure combination, DPC) [5,6], length of hospital stay (LOS), outcome of admission and route of referral from Niigata University Hospital were obtained from the hospital information system. In the DPC system, patient discharge is classified into 16 major diagnostic categories and 1,727 case-mix groups. Data on average and standard deviation (SD) of LOS of the patients in Japan, who were assigned to each DPC, were obtained from the Ministry of Health, Labour and Welfare. Data on age-structured population per district were obtained from National Census of Japan conducted by the Ministry of Internal Affairs and Communications in 2000.

Data on population per district were converted to data on population per 2 km mesh using the GIS software ArcView GIS 3.1a and ArcGIS 9 (Environment Systems Research Institute Inc., CA, USA). Data on locations of paediatric clinics and hospitals with paediatric wards were obtained from lists of the Japan Paediatric Association, Japanese Society of Paediatrics, Japan Medical Association and their local organizations. Road network data also contain codes for different classes of roads, average car speed and rules of the roads in order to calculate how long it would take to drive along a particular road segment [7].

### Referral from clinics and other hospitals

To study the geographic distribution of patients admitted to Niigata University Hospital, empirical Bayesian estimates for the rate of inpatients per childhood population at risk were plotted on a GIS map with 2 km meshes [8]. To study the relationship between spatial accessibility to Niigata University Hospital and the distribution of patients, the number of inpatients who lived within an area that is a certain time by car from Niigata University Hospital was calculated by using Network Analyst (ESRI Japan). The same procedure was performed for other hospitals in Niigata Prefecture in order to compare the inpatient service areas of Niigata University Hospital and general hospitals. The general hospitals had 177 to 312 inpatient beds and one or two paediatricians. Residence records for the patients admitted to the five hospitals in Niigata City were obtained from annual reports published by the hospitals. The distribution of patients referred from other medical facilities was plotted on GIS maps using the kernel density method [9,10] and was compared with the distribution of paediatricians. For the kernel density method, we computed a density layer using a one-kilometre service area radius and a 200-meter-square area as a cell size, which yields a smooth map resolution. To differentiate the patients who have more complex conditions and those who have not, the patients were subdivided into two groups according to the information on DPC of each patient: one group of patients who had severe complications and one group of patients who did not have severe complications. The patients were also subdivided into those who admitted through emergency or taken to the hospital by ambulance and those who were not.

### Referral to aftercare

For the purpose of this study, aftercare was defined as referral to a paediatric aftercare program, such as outpatient care and transfer to paediatric wards of other hospitals, not including foster care, a group home or a nursing home [11]. Distributions of patients referred to the Outpatient Department of Niigata University Hospital, patients referred to outpatient care of other medical facilities and patients transferred to paediatric wards of other hospitals were plotted on GIS maps. Since delay in referral of a patient to aftercare prolongs LOS of the patient, spatial differences in LOS of inpatients might reflect the spatial differences in accessibility to aftercare. To estimate the spatial differences in accessibility to aftercare, LOS of each patient was scored and plotted on a GIS map. The SD scores for LOS (LOSSDS) of a patient who was assigned to a DPC were calculated as follows:

((actual LOS of the patient)−(average LOS of the DPC))(SD of the LOS of the DPC).
 MathType@MTEF@5@5@+=feaafiart1ev1aaatCvAUfKttLearuWrP9MDH5MBPbIqV92AaeXatLxBI9gBaebbnrfifHhDYfgasaacH8akY=wiFfYdH8Gipec8Eeeu0xXdbba9frFj0=OqFfea0dXdd9vqai=hGuQ8kuc9pgc9s8qqaq=dirpe0xb9q8qiLsFr0=vr0=vr0dc8meaabaqaciaacaGaaeqabaqabeGadaaakeaadaWcaaqaaiabcIcaOiabcIcaOiabdggaHjabdogaJjabdsha0jabdwha1jabdggaHjabdYgaSjabbccaGiabdYeamjabd+eapjabdofatjabbccaGiabd+gaVjabdAgaMjabbccaGiabdsha0jabdIgaOjabdwgaLjabbccaGiabdchaWjabdggaHjabdsha0jabdMgaPjabdwgaLjabd6gaUjabdsha0jabcMcaPiabgkHiTiabcIcaOiabdggaHjabdAha2jabdwgaLjabdkhaYjabdggaHjabdEgaNjabdwgaLjabbccaGiabdYeamjabd+eapjabdofatjabbccaGiabd+gaVjabdAgaMjabbccaGiabdsha0jabdIgaOjabdwgaLjabbccaGiabdseaejabdcfaqjabdoeadjabcMcaPiabcMcaPaqaaiabcIcaOiabdofatjabdseaejabbccaGiabd+gaVjabdAgaMjabbccaGiabdsha0jabdIgaOjabdwgaLjabbccaGiabdYeamjabd+eapjabdofatjabbccaGiabd+gaVjabdAgaMjabbccaGiabdsha0jabdIgaOjabdwgaLjabbccaGiabdseaejabdcfaqjabdoeadjabcMcaPaaacqGGUaGlaaa@8A1A@

Contour maps of LOSSDS of the patients were produced by interpolation using the ordinary Kringing method [12].

## Results

### Referral from clinics and other hospitals

**Figure 1:**
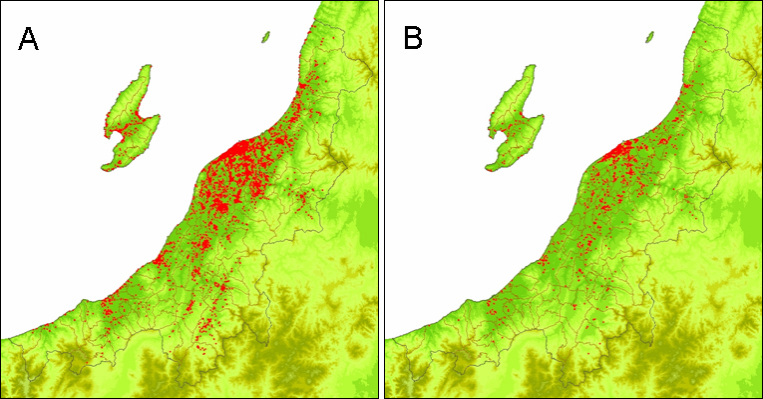
**Distribution of residence of inpatients**. (A) All inpatients (n = 8,177) admitted to Niigata University Hospital during the period from April 2003 to March 2005 were geocoded to a GIS map of Niigata Prefecture. (B) All inpatients under 15 years of age (n = 1,323) were also plotted on a map.

**Figure 2:**
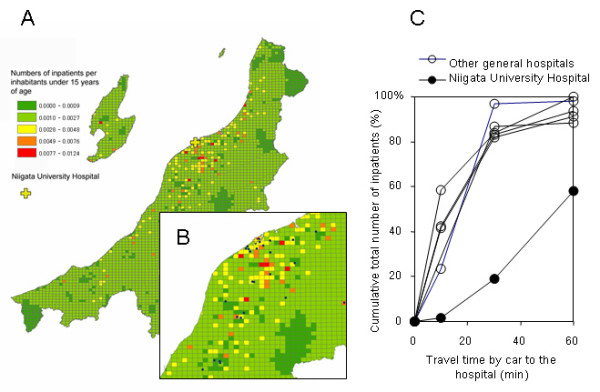
**Medical service area for inpatients**. (A) Empirical Bayesian estimates for the rate of inpatients per childhood population at risk were plotted on a map with 2 km mesh. (B) Magnification of (A). (C) The relationship between travel time by car from Niigata University Hospital (closed circle) or five other hospitals (open circles) in Niigata City and the number of inpatients covered within each area.

During the study period, a total 8,177 patients were admitted to Niigata University Hospital. Among them, 1,323 patients were under 15 years old of age. The outline of the inpatient children and adolescents less than 15 years of age are shown in Table 1. The geographic distributions of all inpatients and inpatients less than 15 years of age were geocoded by residential addresses to a GIS map as shown in Fig. 1. The number of patients who visited Niigata University Hospital without referral from other hospitals and clinics was 800 (60.4%). The empirical Bayesian estimates for the rate of inpatients per childhood population at risk were plotted on a map with 2 km mesh (Fig. 2). Although these patients were predominantly distributed around Niigata City, the capital of Niigata Prefecture, there were some meshes with large numbers of inpatients far from Niigata City (Fig. 2A). This indicated that patients visited Niigata University Hospital from broad area of the prefecture without referral from other medical facilities.

The relationship between spatial accessibility and distribution of residences of patients in Niigata University Hospital and five other hospitals in Niigata City also supports this finding (Fig. 2C). Eighty-three percent of patients admitted to general hospitals lived in an area 30 min from the hospital by car, whereas only 19% of patients admitted to Niigata University Hospital lived in a 30-min area. On the other hand, there were marked spatial differences in the distribution of residences of inpatients that lived in or near Niigata City (Fig. 2B). This suggested that competition with other hospitals to get patients existed in the case of patients who were directly admitted to the hospital without referral from other medical facilities.

**Figure 3:**
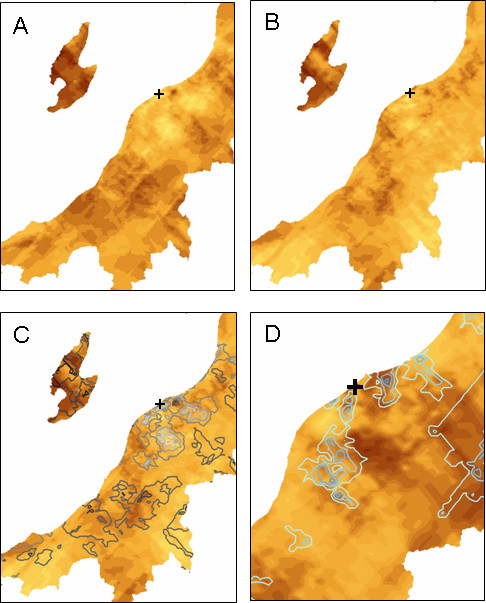
**Referral from clinics and other hospitals**. (A) Kernel density plot of patients referred from clinics and other hospitals. Dark regions represent clustering of these patients. The cross on the map shows the location of Niigata University Hospital. (B) Kernel density plot of the patients who had more complex conditions, i.e., patients who received surgical treatment and patients who had severe complications. (C) Superposition of (A) and (B). The contour represents density shown in (A). (D) Kernel density plot of patients admitted through emergency (filled contour) and patients who were taken to the hospital by ambulance car (contour). Dark contour represents the clusters of patients.

Of the 1,323 patients under 15 years of age, 513 (38.8%) were referred from clinics and other hospitals. Results obtained by using the kernel density method indicated that inpatients that lived near Niigata City tended to be admitted directly to Niigata University Hospital, whereas inpatients who lived far from Niigata University Hospital tended to be referred from other medical facilities (Fig. 3A). the distribution of the inpatients who had more complex conditions was biased in the region far from Niigata University Hospital (Fig. 3B). The distribution of patients who were referred from other medical facilities had a tendency to agree with that of patients who had more complex conditions (Fig. 3C). On the other hand, the distribution of patients admitted through emergency showed a pattern different from that of the distribution of referred patients and that of patients with complex conditions (Fig. 3D).

### Referral to aftercare

**Figure 4:**
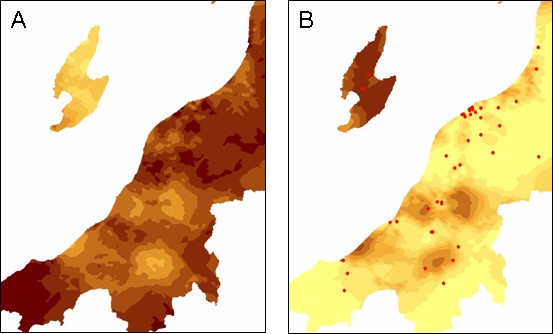
**Referral to aftercare**. (A) Kernel density plot of patients who were referred to the Outpatient Department of Niigata University Hospital. (B) Kernel density plot of patients who were referred to outpatient departments of other hospitals. Dots represent the locations of hospitals with paediatricians.

Almost all of the inpatient children (1275 out of 1323, 96.4%) received referral to aftercare, and 1096 (82.8%) of them received care in Outpatient Department of Niigata University Hospital after discharge. One hundred and fifty-three patients (11.6%) were referred to the clinic or outpatient departments of other hospitals, and 26 patients (2.0%) were transferred to other hospital wards after discharge from Niigata University Hospital. These patients lived far from Niigata City and in the Sado Island, which is located 40 km away from Niigata City (Fig. 4B)

**Figure 5:**
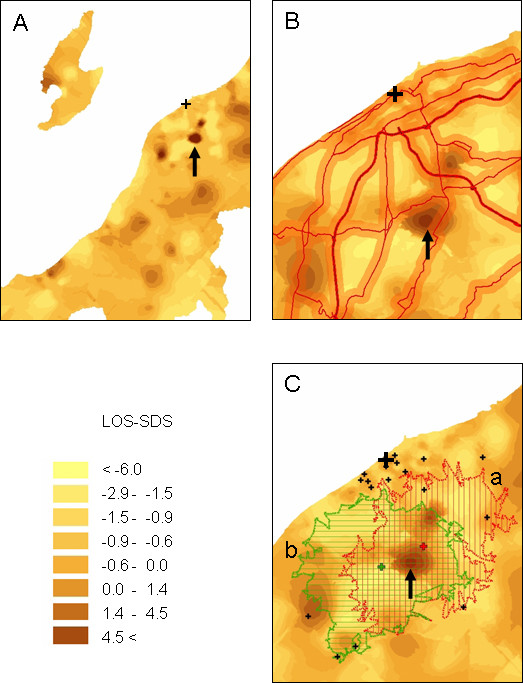
**Clusters of the patients with high LOSSDS**. (A) Contour map of LOSSDS of the patients was produced by interpolation using the ordinary Kringing method. Dark contours represent the clustering of patients with high LOSSDS. Cross and arrow represent the locations of Niigata University Hospital and a focus of clustering of patients with high LOSSDS, respectively. (B) The arterial roads around the focus shown in (A) and their 1,000 meter buffer are shown. (C) Hospitals around the focus shown in (A). The focus is located in an area 30 min by car from the two hospitals a and b. The locations of the two hospitals and their 30-min areas are shown as bold crosses and hatch patterns. The small crosses represent other hospitals.

Next, we investigated spatial difference of LOSSDS in the inpatients in order to ascertain whether there were barriers to referral to aftercare in each district. As shown in Fig. 5A, there were some foci with high LOSSDS near Niigata City (Fig. 5A). We paid special attention to one of the foci (indicated by an arrow) and investigated why the patients who lived in that focus showed high LOSSDS. There were no problems in spatial or physical accessibility in the focus, because the focus was located near arterial roads (Fig. 5B). The focus was located within area 30 min by car from two hospitals (a and b, Fig. 5C). Although these hospitals had beds for children, each hospital had only one paediatrician. Therefore, shortage of paediatric manpower is one of the reasons for the clustering of patients.

## Discussion

The results of our study indicate that more than 60% of inpatients visited a tertiary care hospital in Japan without referral from other medical facilities and that more than 80% of inpatients were referred to the outpatient department of the hospital. Most of the inpatients received outpatient care, inpatient care and aftercare in the same hospital. Patients living near the hospital tended to use the hospital as a secondary care hospital or were admitted without referral from other medical facilities. As a secondary care hospital, the hospital was in competition with other secondary care hospitals to get inpatients. On the other hand, patients who lived far from hospital tended to use the hospital as a tertiary hospital and were referred to the hospital from other medical facilities.

All citizens in Japan are covered by the mandatory health insurance system, the fees for medical service are the same in all medical facilities that provide medical care [5]. There are no general physicians or "gatekeepers" for primary care in Japan. Under these situations, there are no clear differences in care provided by clinics, general hospitals, specialized hospitals, small community hospitals and academic medical centres in Japan. Patients who need primary care often visit hospitals that offer care for specific diseases, and paediatricians working in these hospitals often provide medical care for patients with common diseases. Absence of primary care training, discordance with need of citizens, combined with government-controlled low professional medical fees contribute to an extraordinarily high annual rate of ambulatory patient contact and excessive use of diagnostic testing [13]. Our results showed that even the tertiary care university hospital functions as a secondary care hospital, especially for patients living near the hospital. Moreover, acute care hospitals in Japan usually provide care that is usually provided by nursing homes and home caregivers in other countries such as the USA and UK. The undifferentiated functions of acute care hospitals in Japan make LOS of inpatients extraordinary long compared with that in other advanced countries [3].

Although the care provided by so-called acute care hospitals in Japan is a mixture of various kinds of cares, there are trends toward differentiation and specialization in these hospitals. One such example is the introduction in 2003 of a new inclusive payment system for acute inpatient care in the eighty main hospitals at universities and national centres to replace the traditional fee-for-service payment system [6]. Under the payment system, hospitals can obtain medical fees more efficiently as the LOS decreases. Actually, the LOS of inpatients in these hospitals has become significantly shorter after the introduction of the new payment system [14]. The reduction in LOS makes two new incentives: acquisition of new patients to fill the unoccupied inpatient beds and early discharge to aftercare to shorten the LOS. The former can be accomplished by an increase in referrals from other medical facilities, and the latter needs close partnership between medical facilities participating in aftercare. The results of our study clearly showed spatial differences in partnership between other medical facilities in terms of both referral to the hospital and referral from the hospital. By using GIS, we could easily find districts where the hospital had not established strong partnership with medical facilities. From the results of analysis of LOSSDS, we could get some hint why inpatients that lived in a region stay in the hospital for a long time. The methodology shown in this article is effective to study regions where the partnership is not established and why the partnership is not established in the regions [15,16].

## Conclusion

From our study, it was suggested that the function of university hospital in Japan is unspecialized and that the referral route from the university hospital to aftercare is also unequipped yet.

## Competing interests

The author(s) declare that they have no competing interests.

## List of abbreviations

GIS: geographic information system

LOS: length of hospital stay

DPC: diagnosis procedure combination

LOSSDS: standard deviation score for LOS

## Authors' contributions

ST contributed to concept and design of the article and acquisition and analysis of data. AK supervised all aspects of the study and revised the article. Both of us approved the final version.

## Pre-publication history

The pre-publication history for this paper can be accessed here:



## Figures and Tables

**Table 1:** Characteristic of inpatients. Characteristics of a sample of 1,323 admissions of children and adolescents under 15 years of age from April 2004 to March 2005 at Niigata University Hospital. SD score for LOS was calculated for each patient with a DPC based on the following equation: ((actual LOS of the patient)−(average LOS of the DPC))(SD of the LOS of the DPC).
 MathType@MTEF@5@5@+=feaafiart1ev1aaatCvAUfKttLearuWrP9MDH5MBPbIqV92AaeXatLxBI9gBaebbnrfifHhDYfgasaacH8akY=wiFfYdH8Gipec8Eeeu0xXdbba9frFj0=OqFfea0dXdd9vqai=hGuQ8kuc9pgc9s8qqaq=dirpe0xb9q8qiLsFr0=vr0=vr0dc8meaabaqaciaacaGaaeqabaqabeGadaaakeaadaWcaaqaaiabcIcaOiabcIcaOiabdggaHjabdogaJjabdsha0jabdwha1jabdggaHjabdYgaSjabbccaGiabdYeamjabd+eapjabdofatjabbccaGiabd+gaVjabdAgaMjabbccaGiabdsha0jabdIgaOjabdwgaLjabbccaGiabdchaWjabdggaHjabdsha0jabdMgaPjabdwgaLjabd6gaUjabdsha0jabcMcaPiabgkHiTiabcIcaOiabdggaHjabdAha2jabdwgaLjabdkhaYjabdggaHjabdEgaNjabdwgaLjabbccaGiabdYeamjabd+eapjabdofatjabbccaGiabd+gaVjabdAgaMjabbccaGiabdsha0jabdIgaOjabdwgaLjabbccaGiabdseaejabdcfaqjabdoeadjabcMcaPiabcMcaPaqaaiabcIcaOiabdofatjabdseaejabbccaGiabd+gaVjabdAgaMjabbccaGiabdsha0jabdIgaOjabdwgaLjabbccaGiabdYeamjabd+eapjabdofatjabbccaGiabd+gaVjabdAgaMjabbccaGiabdsha0jabdIgaOjabdwgaLjabbccaGiabdseaejabdcfaqjabdoeadjabcMcaPaaacqGGUaGlaaa@8A1A@

Characteristics	N or mean	%
Age at admission (mean ± SD, years)	4.6 ± 4.9	
Gender, male	709	53.6
SD scores for LOS (mean ± SD)	-0.46 ± 3.66	
Admissions	1,323	
Number of actual patients	1,096	
Referral from other medical facilities	513	38.8
Referral on discharge	1,275	96.4
Outpatient Department of Niigata University Hospital	1,096	82.8
Return to the primary paediatrician	153	11.6
Transfer to paediatric wards of other hospitals	26	2.0
Death during admission	17	1.3
